# A review of social media platform policies that address cannabis promotion, marketing and sales

**DOI:** 10.1186/s13011-023-00546-x

**Published:** 2023-06-19

**Authors:** Carla J. Berg, Cassidy R. LoParco, Yuxian Cui, Alexandria Pannell, Grace Kong, Lynniah Griffith, Katelyn F. Romm, Y. Tony Yang, Yan Wang, Patricia A. Cavazos-Rehg

**Affiliations:** 1 Department of Prevention and Community Health, Milken Institute School of Public Health, George Washington University, 800 22nd St NW, #7000C, Washington, DC 20052 USA; 2 George Washington Cancer Center, George Washington University, 2121 I St NW, Washington, DC 20052 USA; 3 Department of Global Health, Milken Institute School of Public Health, George Washington University, 2121 I St NW, Washington, DC 20052 USA; 4 Department of Psychiatry, Yale School of Medicine, 34 Park Street, New Haven, CT 06519 USA; 5 Wellesley College, 106 Central Street, Wellesley, MA 02481 USA; 6 TSET Health Promotion Research Center, Stephenson Cancer Center; Department of Pediatrics, College of Medicine, University of Oklahoma Health Sciences Center, Oklahoma City, OK 73104 USA; 7 Center for Health Policy and Media Engagement, School of Nursing, George Washington University, 1919 Pennsylvania Ave NW #500, Washington, DC 20006 USA; 8 Department of Psychiatry, Washington University School of Medicine, 660 South Euclid Avenue, Box 8134, St. Louis, MO 63110 USA

**Keywords:** Social media, Marketing, Cannabis, Cannabis industry, Policy

## Abstract

**Background:**

Cannabis marketing exposure via social media may impact use in youth and young adults. Most states with recreational cannabis lack policies regarding social media-based marketing. Thus, we examined such policies among prominent platforms, particularly those popular among youth and young adults.

**Methods:**

In September-October 2022, 3 research team members extracted policies applying to the general community, advertising, and any specific content regarding drug-related content for 11 social media sites: Discord, Facebook, Instagram, Pinterest, Reddit, Snapchat, TikTok, Tumblr, Twitch, Twitter, and YouTube. Using inductive thematic analysis, they then dual-coded restrictions on cannabis-related content (e.g., paid advertising, unpaid promotion, sales). Descriptive analyses were conducted.

**Results:**

Ten (all except TikTok) referenced cannabis/marijuana, 7 (all except Discord, Instagram, TikTok, and YouTube) distinguished different cannabis-derived products, and 5 (Reddit, Snapchat, TikTok, Tumblr, Twitter) noted jurisdictional differences in cannabis regulations/legality. All prohibited sales, 9 (all except Snapchat and Tumblr) prohibited paid advertising, and 4 (Discord, Reddit, Snapchat, TikTok) prohibited unpaid promotion (e.g., user-generated content). All restricted underage access to cannabis-related content. However, policies varied and were ambiguous regarding how “promotion” was defined, whether/how jurisdictional differences in legality were addressed, how businesses may interact on social media, barriers implemented to inhibit the facilitation of sales, and enforcement protocols.

**Conclusions:**

Social media policies regarding cannabis marketing are ambiguous and may facilitate cannabis marketing, promotion, sales, and underage exposure, thus compounding concerns regarding insufficient governmental regulations. Greater specificity in social media cannabis-related policies and enforcement is needed.

**Supplementary Information:**

The online version contains supplementary material available at 10.1186/s13011-023-00546-x.

## Background

As of December 2022, 21 US states have legalized non-medical adult-use (i.e., “recreational”) cannabis [1]. The US cannabis industry hit a record $24 billion in sales in 2021, with expected annual sales of $70 billion by 2028 [2]. One relatively understudied aspect of this new legal cannabis marketplace is cannabis marketing, particularly via social media. Exposure to cannabis marketing on social media is concerning, given the powerful influence of social media as a source of information (and misinformation) about cannabis [3], the increases in social media based cannabis marketing [4–8], high rates of social media use among those underage [9], and associations between cannabis marketing exposure and cannabis use intentions, initiation, and frequency among adolescents [7, 10] and adults [11, 12].

Cannabis-related content can be promoted via various types of social media, including traditional “social network” communities (e.g., Tumblr, Twitter, Facebook), “media sharing networks” (e.g., YouTube, Snapchat, Instagram), and platforms for micro-communities, like gamers (e.g., Discord, Twitch). Cannabis can be marketed via social media through: (1) paid media (e.g., promotion through paid online advertisements), (2) owned media (i.e., promotion through a cannabis brand’s direct channel such as their own website or social media account) [13, 14], and (3) earned media (i.e., promotion through a third party such as consumer- or press-generated content, e.g., social influencers) [13–15].

Because cannabis is a schedule one substance under the federal Controlled Substances Act, federal regulations ban cannabis marketing activities, particularly if they cross state lines, and thus include little, if any, guidance regarding paid advertising in traditional media (e.g., billboards, newspaper) or new media (e.g., online, social media). Federal Trade Commission requirements provide relevant guidance, specifically indicating that advertising must be truthful, evidence-based, and not misleading, and that limitations or disclosures (including those of social influencers) should be clear and conspicuous [16]. Complicating such regulatory considerations further is that, the Agriculture Improvement Act of 2018 allowed hemp-derived CBD to be legally purchased in most states [17] but did not reference other types of cannabis-derived products, like delta-8 THC, delta-10 THC, delta-O THC, and HHC [18], creating ambiguity about their legality under federal law.

At the state level, restrictions on cannabis marketing differ among states with recreational cannabis retail markets. Most states restrict cannabis marketing content (e.g., characteristics that appeal to youth, health claims) and placement (e.g., minimum distances from child-orientated locations) to protect consumers, particularly youth – but few provide guidance regarding online marketing or promotion via social media [19]. Some states have policies modeled after the alcohol industry’s voluntary code, which prohibits companies from advertising in outlets (i.e., on television, radio, print, web) where > 30% of the audience can be “reasonably” expected to be < 21 years old [19, 20], which could apply to social media based marketing. However, this standard is criticized by policymakers and researchers [20], as those ages 12–20 comprise ~ 15% of the US population [21], and thus could overrepresent audiences of outlets where < 30% are < 21 years old. Moreover, the alcohol literature suggests that such policies are ineffective at shielding youth from viewing or interacting with alcohol-related content [21, 22].

It is critical that policies restricting cannabis marketing at the individual platform and jurisdictional levels are appropriate and aligned. Social media platforms have faced increasing scrutiny for their lack of oversight of misinformation and other content that is harmful to public health [23] and have increased their own regulatory efforts regarding paid ads. However, the extent to which such policies pertain to cannabis marketing has not been assessed. This is particularly important given that these retail markets are relatively recent (dating back to 2015) and have rapidly expanded to include a substantial number of states (covering over half of the US population), as well as several countries. This has created a challenging landscape to navigate in general, and may be particularly challenging for social media where geographic/jurisdictional definitions may be unclear. Thus, this study examined policies regarding cannabis promotion and sales on social media platforms popular among youth [9, 13]. Because of the global nature of social media, this study could inform national and international efforts to regulate cannabis marketing on social media.

## Methods

We examined cannabis-related policies on 11 social media platforms that are most popular among US youth: Discord, Facebook and Instagram (together known as Meta), Pinterest, Reddit, Snapchat, TikTok, Tumblr, Twitter, YouTube, and Twitch [9, 13]. Our approach was informed by prior work examining social media policies regarding tobacco marketing [24]. Policies were analyzed using the Framework Method of thematic analysis, which identifies areas of convergence and divergence in qualitative data to identify themes that draw descriptive or explanatory conclusions [25]. First, 3 research team members extracted policies applying to the general community, advertising, and any specific content regarding drug-related content on each social media platform in September-October 2022. Second, to inform our approach to coding, we read each platform’s policies, particularly as the policies pertained to cannabis-related content (e.g., promotion, sales).

Third, we used inductive thematic analysis to code whether: (1) cannabis or marijuana were explicitly addressed in their policies (and whether they noted distinctions from non-THC-containing CBD products or hemp-derived products, e.g., delta-8 THC); (2) the platform indicated jurisdictional considerations regarding legality of cannabis; (3) the following were prohibited or restricted: (a) paid cannabis advertising, (b) unpaid cannabis promotion, and (c) cannabis product sales; and 4) underage access was addressed, and if so: (a) at what age and (b) if cannabis-specific or general guidelines were used (see Table 1 for code definitions and example policy excerpts).

**Table 1:** Codes and Definitions for Social Media Platform Policies Regarding Cannabis Promotion

Codes	Definitions	Examples
Specifies cannabis or marijuana in policy	Whether cannabis or marijuana explicitly addressed in policy (and whether noted distinctions from non-THC-containing CBD products or other product, e.g., delta-8 THC).	“We limit the distribution of or remove some content and accounts, including: individuals and unlicensed retailers offering to sell, purchase or trade alcohol, tobacco, drugs and weapons; offers, attempts, or instructions to bypass purchasing laws and regulation; instructions for creating lethal or toxic substances; and/or commercial sales of marijuana, marijuana products and paraphernalia.” – *Pinterest* “The following applies to campaigns for hemp-derived CBD products: ads cannot make medical, health, or therapeutic claims or show people consuming or smoking CBD products; product being advertised must be non-ingestible, and from legally-derived CBD; ads must be targeted only to states where CBD products are legal; and/or landing pages cannot sell or advertise other products or services prohibited by other Tumblr policies.” – *Tumblr*
Recognizes jurisdictional differences	References to jurisdictional considerations of cannabis legality (e.g., geotargeting to locations where cannabis sales are legal or complying with local laws).	“We allow some limited ads for cannabis, CBD and related products, where legal, with appropriate targeting.” – *Snapchat* “Prohibits ads for illegal or recreational drugs….or anything specifically marketed to aid in the administration or use of such drugs…. The policy doesn’t include… drugs which may be legal or decriminalized in some regions, like marijuana, as long as the ad is properly geotargeted.” – *Tumblr*
Paid advertising restrictions	Restrictions on paid advertisement, including cannabis-containing products, paraphernalia, and cannabis retailers (e.g., dispensaries, consumer lounges).	“Prohibited partners: Partners who engage or attempt to engage in any transaction involving controlled or regulated substances: prohibits the sale, buying, trading, or organizing the distribution of controlled, regulated, or illegal substances through our service (e.g., alcohol, tobacco, vaping products, marijuana, pharmaceuticals without a prescription, narcotics, other drugs).” – *Discord (completely prohibited)* “We allow discussions about the sale of these goods in stores or by online retailers, as well as advocating for changes to regulations of goods and services covered in this policy” – *Facebook (allowed but with restrictions)*
Unpaid promotion restrictions	Restrictions on promotion, including the platform’s restrictions on unpaid content promoting cannabis (e.g., via retailer or brand social media accounts/pages and/or generated by individuals).	“Brick-and-mortar and online retailers may promote firearms, alcohol, and tobacco items available for sale off of our services; however, we restrict visibility of this content for minors. We allow discussions about the sale of these goods in stores or by online retailers, as well as advocating for changes to regulations of goods and services covered in this policy.” – *Facebook (allowed but with restrictions)* “Prohibits the depiction, promotion, or trade of drugs or other controlled substances. Do not post, upload, stream, or share: content that depicts or promotes drugs, drug consumption, or encourages others to make, use, or trade drugs or other controlled substances; content that offers the purchase, sale, trade, or solicitation of drugs or other controlled substances, alcohol or tobacco products.” – *TikTok (completely prohibited)*
Cannabis sales restrictions	Restrictions on sales, including on vendor’s social media account or platform marketplaces, as well as individual user attempts to facilitate sales/purchasing.	“Prohibits its use to conduct illegal behavior or to buy, sell, trade, or share instructions to manufacture any types of drugs, substances, devices, goods or weapons which may be restricted.” – *Tumblr* “Prohibits use of Twitter for any unlawful purpose or in furtherance of illegal activities, including selling, buying, or facilitating transactions in illegal goods or services, as well as certain types of regulated goods or services (e.g., drugs and controlled substances).” – *Twitter*
Underage restrictions	Restriction on underage access, including restricting access to or circulation of cannabis-related content among minors. Additional codes: (a) age specified; and (b) use of cannabis-specific or general guidelines.	“Some restricted goods require minimum age restrictions to comply with Instagram policies, including: alcohol, online pharmacies, prescriptions, drug and alcohol addiction treatment, etc.” – *Instagram (unspecified age)* *“*Adult content should not be made available to minors (< 18).” – *Discord (specifies ≤ 18)* “Any advertising related to the sale of cannabis or general promotion of cannabis brands must be (i) approved in advance by Tumblr, (ii) sold through our direct-sales team (i.e. not programmatic), and (iii) geo-targeted exclusively to people of legal consumption age in the above states (that’s 21 + in each case). Regardless of age-targeting, cannabis ads must not be designed, or appear to be designed, to appeal to under-age purchasers.” – *Tumblr (specifies ≤ 21)*

*Note*: Searches included cannabis, marijuana, and other more general language (drugs, illegal drugs, illegal behavior, etc.).

Qualitative data were organized and analyzed using Excel [26]. Three co-authors (CJB, CL, and YC) dual-coded policies for 3 platforms, discussed and resolved all discrepancies, and revised the coding strategy as needed. Then, all platform policies were dual-coded (Kappas > 0.80), any discrepancies were resolved, and data were charted to facilitate comparison and interpretation. Descriptive data analyses were conducted using SPSS v26.0.

## Results

Table 2 provides categorical codes summarizing each platform’s policies related to cannabis advertising, promotion, and sales. Supplementary Table 1 includes more extensive information regarding the social media platforms’ policies.

**Table 2:** Summary of Social Media Platform Policies Regarding Cannabis Promotion, as of October-November 2022

			Specifies cannabis	Recognizes jurisdictional differences	Paid advertising		Unpaid promotion		Cannabis sales		Underage restrictions					
					Completely prohibited	Allowed but restricted	Completely prohibited	Allowed but restricted	Completely prohibited	Allowed but restricted	Age unspecified	< 18 years old	< 21 years old	Addresses cannabis		
Discord			X	-	X	^−^	X	-	X	-	-	X	-	-		
Facebook			X*	-	X	-	^−^	X	X	-	-	X	-	-		
Instagram			X	-	X	-	-	X	X	-	X	-	-	-		
Pinterest			X*	-	X	-	^−^	X	X	-	-	X	-	-		
Reddit			X*	X	X	-	X	-	X	-	-	X	-	-		
Snapchat			X*	X	-	X	X	-	X	-	X	-	-	-		
TikTok			-	X	X	-	X	-	X	-	-	X	-	X		
Tumblr			X*	X	-	X	-	X	X	^−^	-	-	X	X		
Twitch			X*	-	X	-	-	X	X	-	X	-	-	-		
Twitter			X*	X	X	-	-	X	X	-	-	X	-	X		
YouTube			X	-	X	-	-	X	X	-	-	X	-	X		

Notes: See also Supplementary Table 1 for more details. * Differentiates CBD from cannabis containing THC.

### Explicit reference to cannabis/marijuana

Among the 11 platforms, all except TikTok specifically referenced cannabis; TikTok stated that it prohibits “the depiction, promotion, or trade of drugs or other controlled substances.” In addition, 7 (all except Discord, Instagram, TikTok, and YouTube) explicitly addressed distinctions between cannabis and CBD products, and only one (Tumblr) mentioned hemp-derived cannabinoids such as delta-8 or delta-10 THC. Reddit and Tumblr provided particularly detailed guidance in the policies regarding advertising of CBD products (Supplementary Table 1).

### Jurisdictional considerations regarding legality of cannabis

Jurisdictional differences in regulations/legality of cannabis were explicitly noted by 5 social media platforms (Reddit, Snapchat, TikTok, Tumblr, Twitter). For example, Snapchat’s policies stated: “Ads must comply with all applicable laws, statutes, ordinances, rules, public order rules, industry codes, regulations, and cultural sensitivities in each geographic area where the ads will run. We allow some limited ads for cannabis, CBD and related products, where legal, with appropriate targeting”; Tumbler’s policies stated: “The policy [prohibiting advertising] doesn’t include: drugs which may be legal or decriminalized in some regions, like marijuana, as long as the ad is properly geotargeted”. Reddit’s policies stated: “Exceptions to the policy [prohibiting certain types of advertising] include: CBD products in the US and cannabis products in Canada”.

### Paid cannabis advertising

Nine of the 11 platforms (all except Snapchat and Tumblr) had policies stating complete prohibition of paid advertising for cannabis products. As noted above, both Snapchat and Tumblr explicitly allowed cannabis product advertising within jurisdictions where cannabis is legal.

### Unpaid cannabis promotion

Among the 11 platforms, unpaid promotion of cannabis (e.g., via user-generated content) was prohibited among 4 (Discord, Reddit, Snapchat, TikTok); however, these policies were generally brief and/or ambiguous (Supplementary Table 1). For example, Discord’s policies stated: “Do not organize, promote, or engage in any illegal or dangerous behavior, such as selling or facilitating the sale of prohibited or potentially dangerous goods (drugs, controlled substances).” Discord’s policy language prohibited cannabis-related content promotion whether cannabis was legal within the jurisdiction or not, and Reddit, Snapchat, and TikTok recognized jurisdictional differences but also prohibited such content nonetheless.

The other 7 platforms had various restrictions and allowances. Some allowances related to the extent of content and discourse regarding cannabis (e.g., Facebook: “We allow discussions about the sale of these goods in stores or by online retailers, as well as advocating for changes to regulations of goods and services covered in this policy”). Other allowances were made for retailers to engage in the platform (e.g., Instagram prohibits “any marijuana seller, including dispensaries, from promoting their business by providing contact information like phone numbers, email addresses, street addresses, or by using the “contact us” tab in Instagram Business Accounts. However, we do allow people to include a website link in their bio information”). Twitch stated that it prohibits “discussing or broadcasting [drug-related] topics in a way that glorifies, promotes, or encourages” drug use.

### Cannabis sales

While all 11 platforms general referenced prohibiting cannabis sales, there was variability regarding their exact policies and restrictions. Most stated that using the platform to facilitate cannabis sales or purchases were prohibited. However, the extent of barriers to impede attempts to facilitate sales varied. For example, some platform policies (e.g., Facebook, Instagram) explicitly indicated that retailers were allowed to have a profile/account, while most others’ policies were vague in this regard or did not explicitly address parameters around retailers/manufacturers engagement in their platforms through profiles/accounts.

Some platforms had policy language regarding types of prohibited content that was seemingly contradictory. For instance, YouTube indicated: “This content will earn no ad revenue: reviews of cannabis coffee shops, head shops, dealers, dispensary tours, etc.; and selling or buying drugs online or offline (sharing links to drug purchasing sites or the physical addresses of drug purchasing locations)”. However, elsewhere YouTube’s policies state that it prohibits posts “aiming to directly sell, link to, or facilitate access to controlled narcotics and other drugs; making the sale of these items or facilitating the use of these services possible by posting links, email, phone number or other means to contact a seller directly; or featuring drugs with the goal of selling them”.

### Underage access to cannabis-related content

All platforms had some mention of restricted access to cannabis-related content among minors or underage individuals. Three (Instagram, Snapchat, Twitch) had general language referencing minors or underage individuals, 7 (Discord, Facebook, Pinterest, Reddit, TikTok, Twitter, YouTube) indicated a minimum age threshold of 18, and one (Tumblr) indicated a minimum age threshold of 21.

Seven had general (not cannabis-specific) age restrictions (Discord, Facebook, Instagram, Pinterest, Reddit, Snapchat, Twitch). For example, Discord’s policies stated: “Adult content should not be made available to minors (< 18)”, and Snapchat’s policies stated: “All ads must be suitable for their selected audience in each geographic area where the ads will run.” Four platforms (Tiktok, Tumblr, Twitter, YouTube) had age restrictions that specifically referenced cannabis; for example, Tumblr’s policies stated: “cannabis ads must not be designed, or appear to be designed, to appeal to under-age purchasers.” YouTube’s policies indicated that it prohibits “content that endangers the emotional and physical well-being of minors (< 18 in most countries/regions)” which is determined by considering “whether: the content promotes a product that contains drugs, nicotine, or a controlled substance; the upload is educational, documentary, scientific or artistic in nature; and there’s any commentary discouraging the act” (among other factors).

## Discussion

Findings from the current study highlight the ubiquitous prohibition of cannabis sales on 11 social media platforms (as of October-November 2022), but less consistency with regard to cannabis promotion and limited explicit language in terms of their restrictions or their enforcement protocols. For example, 9 (all except Snapchat and Tumblr) prohibited paid cannabis product advertising, and 4 (Discord, Reddit, Snapchat, TikTok) prohibited unpaid cannabis promotion (e.g., via user-generated content). All but one platform explicitly referenced cannabis, 7 explicitly addressed distinctions between cannabis and CBD products, and 5 noted jurisdictional differences in regulations/legality of cannabis (albeit often unclear how “illegal” was determined, e.g., whether state/local policies are considered).

The individual platforms varied in terms of their level of sophistication related to regulating cannabis-related content. Tumblr was among the most sophisticated, specifying cannabis, distinguishing CBD and other hemp-derived products, accommodating advertising within the context of jurisdictional differences, and indicating the minimum age of 21 in age restrictions. Reddit, Snapchat, TikTok, and Twitter also recognized jurisdictional differences but varied in terms of their allowances for paid and unpaid cannabis promotion. Facebook, Instagram, Pinterest, Twitch, and YouTube were similar, specifying cannabis in its policies, but not recognizing jurisdictional differences, prohibiting paid advertising and sales, but allowing some unpaid promotion.

Policies prohibiting paid cannabis advertising and sales (e.g., business-to-customer, user-to-user) were relatively clear. However, having a policy does not guarantee its enforcement, and violation of platform policies is common [24]. Moreover, platform policies varied and were ambiguous in terms of how “promotion” was defined (e.g., including sponsored/influencer activity), how businesses can interact on social media, activities such as gifting, and oversight and enforcement protocols. These types of activities underscore the need for monitoring unpaid promotional content posted by businesses. Additionally, some platforms (e.g., YouTube) had policy language that was contradictory with itself, implying that promotional content could be posted but not for revenue [27, 28].

User-generated content is also a concern given the potential impact on other platform users [7]. Platform policies generally prohibited cannabis or illegal drug promotion. However, there was minimal guidance regarding the nature of prohibited cannabis-related posts and interactions. Unsurprisingly, social media content that promotes/encourages cannabis use is highly prevalent and may entail using covert mechanisms (e.g., code words) to conceal cannabis-related content [5, 7]. For example, there were 37 million posts with #420 (referencing time for cannabis use) on Instagram as of December 18, 2022 (per direct observation). Additionally, analyses of YouTube content documented extensive presence of content portraying use, alongside celebrity endorsements, product reviews, and use instructions [28, 29], and only one-fifth were age restricted [28]. Thus, it is crucial that cannabis-related promotion policies, including those related to enforcement, are more detailed and explicit.

All platforms had some mention of restricted access to cannabis-related content among minors or underage individuals. While underage access can be restricted through age-gating, research on tobacco-related age-gating suggests that it can easily be bypassed (e.g., by misreporting age) and is not effectively enforced on social media platforms [30–32]. Although age verification methods on social media platforms are evolving [33], their effectiveness is unclear. Another concern related to underage access is that only one platform (Tumblr) indicated a minimum age of 21 for exposure to cannabis-related content, which also applied to alcohol and tobacco, despite the legal age for all 3 being 21. Thus, social media platforms should consider restricting access based on legal age and identifying more effective age verification strategies and enforcement protocols.

Another related concern is exposure to cannabis promotion among those in jurisdictions where cannabis is illegal. Monitoring paid content on social media poses challenges for regulatory agencies for various reasons, including sheer volume and identification of the source’s geographical location. Moreover, sponsored posts made by influencers and indirect promotion via political messaging are increasingly common [34–36], and sponsored posts do not always disclose financial relationships or make use of official branded post tools that make them easily identifiable [30, 31]. Thus, these types of communications are difficult to monitor and regulate, both by social media platforms and by regulatory agencies who could exercise their purview over advertising on social media, including federal oversight when advertising crosses state borders. Because of the myriad of complexities related to monitoring and surveillance, social media platforms and federal agencies must develop more effective strategies for automatically identifying cannabis-related content. More specifically, such strategies should leverage the potential of artificial intelligence [37] or machine learning [38], as well as geo- and age-targeting controls, to more proactively prevent exposure among those who are underage or in jurisdictions where cannabis is illegal.

### Study limitations

The social media platforms included in this study do not represent all social media platforms. However, this study focused on 11 platforms most popular among US youth and young adults Twitch [9, 13], thus presenting those platforms where cannabis products may be most likely marketed and age verification related issues are most relevant. Second, this study did not account for historical platform policy context, as reliable sources of prior policies do not exist. This limitation does not undermine findings from this analysis, which provided an extensive overview of the most current platform policies (Supplementary Table 1) and critically reviewed and compared the current cannabis-related policies across platforms (Table 2). However, this limitation does highlight the need for transparency from social media companies regarding their policies and changes in policies over time.

### Implications for practice and research

Social media platforms must adopt clearer, more proactive policies and enforcement protocols to restrict the promotion of cannabis and other drugs and prevent exposure among those who are underage. Federal regulations should also require social media to enact policies to restrict the promotion of all drugs. Precedent for such action exists, as the recent U.S. Surgeon General Advisory highlighted steps that social media platforms can take to protect the public from misinformation during the COVID-19 pandemic [23], which could apply to cannabis. For example, social media platforms should: monitor, assess, and take responsibility for the harms of cannabis promotion, both by industry and by individual users, on their platforms; offer researchers open access to real-time and historical data regarding their policies and social media content to examine cannabis promotion and its impact; determine what internal policies and algorithms may contribute to cannabis promotion; publish their protocols for monitoring social media content and the removal of content that is noncompliant with their policies; and increase information from accurate sources about potential cannabis-related risks [23]. Such strategies might increase accountability among social media platforms and engage researchers in the pursuit of enhancing protocols for detecting and reducing harmful cannabis-related content circulation on social media.

In addition, future research should leverage available data to monitor the extent to which social media platforms are enforcing their existing policies. For instance, prior research has compared online content with state advertising policies to highlight enforcement gaps [39]. This work will inform efforts to hold social media platforms accountable and to estimate the impact of different policy and enforcement strategies.

## Conclusions

Given the vast use of social media globally [13], particularly among young people [9], it is critical to reduce the potential negative impacts of cannabis promotion on social media. The current study documented the variability, ambiguities, and contradictory language in social media platform policies regarding cannabis sales/promotion, which may catalyze the cannabis industry to exploit social media as a strategic marketing channel given its low-cost and high reach, especially among young people [9, 13], and its potential to influence adolescent [7, 10] and adult behaviors [11, 12]. Thus, platform policies should explicitly prohibit cannabis promotion/sales (both direct and indirect methods, e.g., influencers) and underage access to cannabis promotional content, and detail and communicate their enforcement protocols. Furthermore, future research should assess policy implementation, enforcement, and compliance across platforms, focusing on preventing underage access and access beyond legal jurisdictions. Finally, given the rapid pace of cannabis legislation, researchers and regulators (both within jurisdictions and social media platforms) must continually assess the landscape of cannabis-related policies of social media platforms and evolve to keep pace with the cannabis retail environment and its reflection on social media.

## Electronic supplementary material

Below is the link to the electronic supplementary material.

Supplementary Material 1

## Data Availability

All data generated or analyzed during this study are included in this published article and its supplementary information files.
