# Supplementary material for: A review of social media platform policies that address cannabis promotion, marketing and sales
Source: Subst Abuse Treat Prev Policy. 2023 Jun 19;18:35. doi: 10.1186/s13011-023-00546-x (PMC10278361; doi:10.1186/s13011-023-00546-x)
Supplement: Supplementary file 1 — Supplementary Material 1 [file 13011_2023_546_MOESM1_ESM.docx]

| **Supplementary Table 1. Social Media Platform Policies Regarding Cannabis Promotion, as of October-November 2022** | | | |
| --- | --- | --- | --- |
|  | **General Community Guidelines** | **Policies Regarding Paid Advertising *** | **Age Restrictions **** |
| **Discord** | **Do not organize, promote, or engage in any illegal or dangerous behavior,** such as selling or facilitating the sale of prohibited or potentially dangerous goods (drugs, controlled substances).  URL: <https://discord.com/guidelines> | **General guidelines**: Prohibited partners: 1) Partners who engage or attempt to engage in any transaction involving controlled or regulated substances: prohibits the sale, buying, trading, or organizing the distribution of controlled, regulated, or illegal substances through our service (e.g., alcohol, tobacco, vaping products, marijuana, pharmaceuticals without a prescription, narcotics, other drugs). Discussion of legal, controlled substances is allowed in age-restricted spaces. 2) Partners who promote pharmaceutical products or specific healthcare services, such as diet products: prohibits the promotion of all pharmaceutical products and services, and some healthcare products and services. Restrictions are based on the product or service being promoted, as well as the country the campaign is targeting.  URL: <https://support.discord.com/hc/en-us/articles/4413237398935-Discord-s-Brand-Partnership-Guidelines> | Adult content should not be made available to minors (<18).  URL: <https://discord.com/guidelines>  URL: <https://support.discord.com/hc/en-us/articles/4413237398935-Discord-s-Brand-Partnership-Guidelines> |
| **Facebook** | Prohibits attempts by individuals, manufacturers, and retailers to purchase, sell, raffle, gift, transfer or trade certain goods and services on our platform; and content that attempts to buy, sell, trade, donate or gift or asks for marijuana.  URL: <https://transparency.fb.com/policies/community-standards/regulated-goods/> | **General guidelines**: Prohibits attempts by individuals, manufacturers, and retailers to purchase, sell, raffle, gift, transfer or trade certain goods and services on our platform. Brick-and-mortar and online retailers may promote firearms, alcohol, and tobacco items available for sale off of our services; however, we restrict visibility of this content for minors. We allow discussions about the sale of these goods in stores or by online retailers, as well as advocating for changes to regulations of goods and services covered in this policy. Do not post content that: attempts to buy, sell, trade, coordinate the trade of, donate, gift or asks for non-medical drugs; admits to buying, trading or coordinating the trade of non-medical drugs by the poster of the content by themselves or through others; admits to personal use without acknowledgment of or reference to recovery, treatment, or other assistance to combat usage – this content may not speak positively about, encourage use of, coordinate or provide instructions to make or use non-medical drugs; and/or coordinates or promotes (speaks positively about, encourages use of, or provides instructions to use or make) non-medical drugs. Ads must not: violate Community Standards; promote the sale or use of illicit or recreational drugs, or other unsafe substances, products or supplements, as determined by Meta at its sole discretion; promote or offer the sale of THC products or cannabis products containing related psychoactive components; or promote or offer the sale of cannabidiol (CBD) or similar cannabinoid products. Ads that promote or offer the sale of Hemp products must comply with all applicable local laws, required or established industry codes and guidelines.  **Monetized content**: Soliciting engagement behaviors cannot be monetized if they have content that requests compensation in exchange for extreme behavior, such as consumption of drugs, tobacco, or alcohol.  **Branded content:** Certain goods, services, or brands may not be promoted with branded content. We prohibit promotion of illegal products or services, tobacco products, vaporizers, electronic cigarettes, or any other products that simulate smoking, drugs and drug-related products, including illegal or recreational drugs.  **Unsafe substances**: Advertisers can’t run ads that promote the sale or use of illicit or recreational drugs, or other unsafe substances, products or supplements. *Note*: This policy prohibits the sale or use of illicit or recreational drugs, or other unsafe substances, products or supplements, including prescription drugs. Ads can't: solicit, buy, sell, trade, donate or gift (such as freebies) illicit, recreational, or other potentially unsafe drugs, products or supplements; encourage the consumption of illicit, recreational, or other potentially unsafe drugs, products or supplements; or promote the sale of drug-related paraphernalia, such as bongs, rolling papers and vaporized delivery devices. avoid using images: of smoking-related accessories (like bongs and rolling papers); that imply the use of a recreational drug; and/or of either recreational or medical marijuana. Ads can: refer to prohibited drugs, products, or supplements for the purposes of political advocacy, news, and awareness campaigns as long as they don’t promote the sale or consumption of those substances  **Prescription drugs**: Written permission required, no targeting those under 18, restricted to allowable countries.  URL: <https://transparency.fb.com/policies/community-standards/regulated-goods/>  URL: <https://transparency.fb.com/policies/ad-standards>  URL:  <https://www.facebook.com/business/help/1348682518563619?id=2520940424820218&helpref=search&sr=5&query=drug>  URL:  <https://www.facebook.com/business/help/221149188908254?helpref=search&sr=2&query=drug>  URL: <https://transparency.fb.com/policies/ad-standards/dangerous-content/unsafe-supplements/>  URL: <https://www.facebook.com/business/help/263390265553560?id=434838534925385> | Prohibits tobacco/alcohol advertising to minors (<18).  URL: <https://transparency.fb.com/policies/community-standards/regulated-goods/> |
| **Instagram** | Prohibits buying or selling firearms, alcohol, and tobacco products between private individuals, and buying or selling non-medical or pharmaceutical drugs. Also removes content that attempts to trade, co-ordinate the trade of, donate, gift, or ask for non-medical drugs, as well as content that either admits to personal use (unless in the recovery context) or coordinates or promotes the use of non-medical drugs.  URL: <https://help.instagram.com/477434105621119> | **Marijuana**: Prohibits people or organizations to use the platform to advertise or sell marijuana, regardless of the seller’s state or country. Prohibits any marijuana seller, including dispensaries, from promoting their business by providing contact information like phone numbers, email addresses, street addresses, or by using the “contact us” tab in Instagram Business Accounts. However, allows people to include a website link in their bio information.  **For branded content:** Prohibits promotion of: illegal products or services, tobacco products, vaporizers, e-cigarettes, or any other products that simulate smoking; and/or drugs and drug-related products, including illegal or recreational drugs.  **For monetized content:** Prohibits content that requests compensation in exchange for extreme behavior, such as: consumption of inedible substances; consumption of drugs, tobacco, or alcohol.  URL: <https://www.facebook.com/help/instagram/789164081427334>  URL: <https://help.instagram.com/1695974997209192/>  URL: <https://help.instagram.com/2635536099905516> | Some restricted goods require minimum age restrictions to comply with [Instagram policies](https://l.instagram.com/?u=https%3A%2F%2Fwww.facebook.com%2Fbusiness%2Fhelp%2F221149188908254%3Fhelpref%3Dfaq_content&e=AT1fcobu9Q9B1_mWxcPH_g28RC3md5SZzBqiIEzjn8o_jh7D-n-5XVgtaRyJl05Ted5aetwaKwCaLXr1MwlV_DqAj6PA2KhdjU5XjZxwBT5kap6Tv_A4ZZUZWMkFEvxBLJ9pMrgNgKr8VBhaySrJzA), including: alcohol, online pharmacies, prescriptions, drug and alcohol addiction treatment, etc.  Users can choose to set up a default minimum age that will be applied everywhere, or users can set up country-specific minimum ages to follow local policies.  URL:<https://help.instagram.com/316932422966736/?helpref=search&query=drugs&search_session_id=f0a5099ff35c8f886828a7af1d50084f&sr=1>  URL: <https://www.facebook.com/business/help/221149188908254?helpref=faq_content>  URL:  <https://help.instagram.com/853772598370828/?helpref=search&query=drug%20age&search_session_id=524d8ce0497fd1e7cc89c7105418e83d&sr=5> |
| **Pinterest** | We limit the distribution of or remove some content and accounts, including: individuals and unlicensed retailers offering to sell, purchase or trade alcohol, tobacco, drugs and weapons; offers, attempts, or instructions to bypass purchasing laws and regulation; instructions for creating lethal or toxic substances; and/or commercial sales of marijuana, marijuana products and paraphernalia.  URL: <https://policy.pinterest.com/en/advertising-guidelines> | **Drugs:** Prohibits advertising of: the sale or use of illegal or recreational drugs; informational material about the use or legalization of illegal or recreational drugs; ads including imagery of illegal or recreational drugs; accessories associated with drug use including paraphernalia for using, storing or consuming illegal or recreational drugs; and/or products containing CBD or similar compounds. We will allow ads for topical hemp seed oil products in the US that contain negligible amounts of THC and no CBD and make no therapeutic or medicinal claims.  URL: <https://policy.pinterest.com/en/merchant-guidelines> | Prohibits ads targeting any audience based on various characteristics (e.g., age, race, color, or ethnic origin). Explicitly prohibits ads to Pinners under 18 years old.  URL: <https://policy.pinterest.com/en/advertising-guidelines> |
| **Reddit** | “Keep it legal, and avoid posting illegal content or soliciting or facilitating illegal or prohibited transactions.”  Prohibits its use to solicit or facilitate any transaction or gift involving certain goods and services, including: drugs, including alcohol and tobacco, or any controlled substances (except ads placed in accordance with our advertising policy).  URL: <https://www.redditinc.com/policies/content-policy>  URL: <https://www.reddithelp.com/hc/en-us/articles/360043513471> | **Creative and ad targeting prohibitions:** Prohibits ads that: 1) make unrealistic, exaggerated, unsubstantiated claims about the effectiveness of a drug or supplement; 2) make guarantees (e.g., implying, suggesting, claiming that the drug or supplement being advertised is guaranteed or needed to cure an ailment or improve one’s abilities); 3) make claims there are no side-effects or that they cannot cause harm; 4) use imagery or text on the ad or landing page that appeal to minors; 5) suggest that one must take a drug or supplement; 6) target minors, sensitive groups, or support related subreddits; and/or 7) serve ads outside of the country that they are located in. (Elsewhere stated: Advertisers may not advertise products or services in a location where those products and services are illegal.)  **Location-specific policy compliance:** Advertisers may not advertise products or services in a location where those products and services are illegal.  **Recreational drugs: R**estricts ads promoting the use, sale, manufacturing, and branding of recreational drugs and other related products and services. **Prohibited recreational drugs (e.g., c**ocaine, cannabis, methamphetamine, psilocybin mushrooms) **and related products and services;** Drug paraphernalia (e.g., bongs, pipes, grinders, clothing); Products that facilitate, or are associated with, the production of recreational drugs; Facilities that produce recreational and illicit drugs; Cannabis dispensaries; Events and research sponsored recreational drug producers; and/or Rehab facilities. **There are two exceptions to this policy: *CBD (US only):*** Allows ads for topical and non-ingestible hemp-derived CBD products in US only. Advertisers must be managed by a Reddit Sales rep and ads must be pre-approved; may only advertise non-ingestible CBD products intended for topical use, containing hemp-derived CBD that has been legally grown, extracted, processed, and manufactured; and may not target states where topical CBD products remain illegal (Hawaii, Idaho, Kansas, Mississippi, Missouri, and South Dakota). Advertisers may only target users aged ≥21; Ad landing pages cannot advertise or sell products or services prohibited by other Reddit policies; Ads and product landing pages cannot make health, medical, or therapeutic claims; and Advertisers and any products advertised must be compliant with any federal, state, and/or local laws, regulations, and/or rules applicable in the jurisdiction in which they are targeting (including but not limited to any laws, regulations, and/or rules relating to labeling, testing, manufacturing, product registration, licensing, and/or permitting). ***Cannabis (Canada Only):*** Allows ads for cannabis products and services in Canada only. Advertisers must be managed by a Reddit Sales rep and ads must be pre-approved; Advertisers must have a license from Health Canada and may only target provinces where they are licensed to operate; The direct sale of cannabis is not permitted. Ads can only promote and bring awareness to the brand and its products; and Ads and ad landing pages cannot make health, medical, or therapeutic claims, appeal to people under age 18, vulnerable or sensitive groups, discuss prices or distribution, or display reckless, dangerous, or otherwise irresponsible behavior. Advertisers and any products or services advertised must be compliant with any federal, provincial, and/or local laws, regulations, and/or rules applicable in the jurisdiction in which they are targeting.  **Pharmaceutical drugs:** Ads that promote over-the-counter pharmaceutical drugs, drug manufacturers, or medical devices are permitted on Reddit. All advertisers promoting over-the-counter pharmaceutical drugs, drug manufacturers, or medical devices must be approved by the FDA or its international equivalent and managed by a Reddit Sales Representative. Ads for prescription pharmaceutical drugs are not allowed on Reddit, except in the US and Canada.  URL: <https://redditinc.force.com/helpcenter/s/article/Reddit-Advertising-Policy-Restricted-Advertisements#N10>  URL: <https://redditinc.force.com/helpcenter/s/article/Reddit-Advertising-Policy-Targeting-Guidelines> | Advertisers may not target minors (<18) or use imagery or text on the ad or landing page that appeal to minors; and/or targeting minors, sensitive groups, or support related subreddits.  URL: <https://redditinc.force.com/helpcenter/s/article/Reddit-Advertising-Policy-Targeting-Guidelines>  URL: <https://redditinc.force.com/helpcenter/s/article/Reddit-Advertising-Policy-Restricted-Advertisements> |
| **Snapchat** | Prohibits: its use for any illegal activity, including…to buy, sell or facilitate sales of illegal or regulated drugs; and promotion of regulated goods or industries, including illegal gambling, tobacco products, and alcohol.  URL: <https://values.snap.com/privacy/transparency/community-guidelines> | **Drugs:** Ads must comply with all applicable laws, statutes, ordinances, rules, public order rules, industry codes, regulations, and cultural sensitivities in each geographic area where the ads will run. Prohibits ads that facilitate or encourage illegal activity (conduct, products, or enterprises). For example, ads should not depict illegal drug use. Prohibits the depiction of illegal drug use or the recreational use of pharmaceuticals. We allow some limited ads for cannabis, CBD and related products, where legal, with appropriate targeting. We do not allow the depiction of smoking or vaping, except in the context of public health messaging or smoking cessation.  **Prescription medicines:** All advertisers of prescription medicines must be pre-approved by Snap and may be required to submit proof of authorization to advertise the medicine in the applicable jurisdiction.  **Deceptive content:** Prohibits ads that are false or misleading, including deceptive claims, offers, functionality, or business practices….  URL: <https://www.snap.com/en-US/ad-policies#general-requirements> | All ads must be suitable for their selected audience in each geographic area where the ads will run.  URL: <https://www.snap.com/en-US/ad-policies#general-requirements> |
| **TikTok** | Prohibits the depiction, promotion, or trade of drugs or other controlled substances. Do not post, upload, stream, or share: content that depicts or promotes drugs, drug consumption, or encourages others to make, use, or trade drugs or other controlled substances; content that offers the purchase, sale, trade, or solicitation of drugs or other controlled substances, alcohol or tobacco products (including vaping products, smokeless or combustible tobacco products, synthetic nicotine products, E-cigarettes, and other ENDS [Electronic Nicotine Delivery Systems]); content that provides information on how to buy illegal or controlled substances; and/or content that depicts or promotes the misuse of legal substances, or instruction on how to make homemade substances, in an effort to become intoxicated.  URL: <https://www.tiktok.com/community-guidelines?lang=en#32> | **General guidelines:** Prohibited products or services: promotion, sale, solicitation of, or facilitation of access to illegal drugs, controlled drugs, prescription drugs (prohibited in some markets), drugs for the purpose of recreation, homeopathy, enhancement, performance, including weight loss; promotion, sale, solicitation of, or facilitation of access to drug paraphernalia, or accessories or supplies any of such; promotion of or facilitation of access to unauthorized drugstores, pharmacies, or dispensaries; depiction of or featuring drug use, drug abuse or prescription drug abuse; and depiction of or featuring drugs-related words, symbols, or images, whether in the form of visual or audio content, or any of such.  **Drugs, tobacco and related paraphernalia:** Ad creatives & landing page must not display or promote illegal drugs, controlled drugs, prescription drug abuse, recreational drugs, drug paraphernalia, or accessories or supplies any of such, including the use of them – or promote tobacco, tobacco-related products such as cigars, tobacco pipes, rolling papers, or e-cigarettes, or smoking-related behavior in real life, including but not limited to alternatives which imitate the act of smoking.  **Pharmaceuticals, healthcare, medicines:** Ads for pharmaceuticals, healthcare, and medicines must target 18+ users and comply with local laws of the target country. Over-the-counter or prescription drugs must be approved by the regional regulating authority and may not contain misleading or inaccurate claims. We do allow ads for brick and mortar pharmacies and online pharmacies that are certified by a third-party licensing organization. Proof of licensing may be necessary for approval.  **Media and entertainment:** Ads that promote films, TV-shows, or games must not contain…drug use.  URL:  [https://ads.tiktok.com/help/article/tiktok-advertising-policies-industry-entry?redirected=1#](https://ads.tiktok.com/help/article/tiktok-advertising-policies-industry-entry?redirected=1)  URL: <https://ads.tiktok.com/help/article/tiktok-advertising-policies-ad-creatives-landing-page?redirected=1>  URL: <https://ads.tiktok.com/help/article/tiktok-advertising-policies-industry-entry-north-america?redirected=1>  URL: <https://ads.tiktok.com/help/article?aid=10005232> | Prohibits content that suggests, depicts, imitates, or promotes harmful activities among minors (<18), including possession or consumption of substances prohibited for minors (e.g., alcohol, tobacco), misuse of legal substances, engagement in illegal activities, or participation in activities, physical challenges, or dares that may threaten the well-being of minors; or that offers instruction targeting minors on how to buy, sell, or trade alcohol, tobacco, or controlled substances.  Ad creatives & landing pages must not display, facilitate or promote inappropriate behaviors involving minors, such as underage drinking or smoking, or influence minors to participate in unsuitable activities.  URL: <https://www.tiktok.com/community-guidelines?lang=en#32>  URL: <https://ads.tiktok.com/help/article/tiktok-advertising-policies-ad-creatives-landing-page?redirected=1> |
| **Tumblr** | Prohibits its use to conduct illegal behavior or to buy, sell, trade, or share instructions to manufacture any types of drugs, substances, devices, goods or weapons which may be restricted.  URL: <https://www.tumblr.com/abuse/drug-sale> | **Location-specific policy compliance:** Follow the law: Always, always, always make sure your advertising practices comply with all laws that are applicable to you and your activities in all relevant jurisdictions**.**  **Illegal drugs:** Prohibits ads for illegal or recreational drugs, even the fun ones, or anything specifically marketed to aid in the administration or use of such drugs. These include: products used to bypass drug tests; pipes, rolling papers, bongs, vaporizers, other smoking vessels (except as otherwise authorized by this policy); substances marketed as “legal highs,” such as the salvia plant, and other stupid and dangerous things; and/or services enabling access to drugs. The policy doesn’t include: news and current events related to drugs and drug paraphernalia; drug rehabilitation programs; drug education and documentary; e-cigarettes, provided the advertising is legal in the target market; and/or drugs which may be legal or decriminalized in some regions, like marijuana, as long as the ad is properly geotargeted. Creative content: Prohibits the portrayal or promotion of illegal drugs and drug paraphernalia in creative content. This applies to references both explicit and implied. For example, creative that implies that a person is under the influence of an illegal drug, even if it's hella funny, is not allowed. What the policy doesn’t apply to: Pharmaceuticals; News and current events related to drugs and drug paraphernalia; Drug rehabilitation programs; and Drug education and documentary.  **Cannabis:** Restricts the promotion and sale of cannabis and cannabis-adjacent products and brands through advertising on its site, with the exception of California, Illinois, Massachusetts, Oregon, Arizona, and Colorado. “Cannabis-adjacent products” include, but are not limited to, water pipes, rolling papers, and Weedmaps. They do not include hemp-derived CBD products. Cannabis-related ads must be: approved in advance by Tumblr; sold through our direct-sales team (i.e. not programmatic); geo-targeted exclusively to people of legal consumption age in the above states (that’s 21+ in each case); and clear that the ad is for cannabis or cannabis-adjacent products. They must include all legally required notices and display the license number of the business (which must be verified). Must also come with disclaimers (e.g., ‘Only intended for consumers in CO, CA, MA, AZ, OR, or IL; not intended for minors. The advertiser is wholly responsible for accuracy and compliance with the law.’); and/or For ads in Oregon, include language materially similar to the following: "Do not operate a vehicle or machinery under the influence of this drug; for use only by adults twenty-one years of age and older; keep out reach of children" .Cannabis-related ads must not: be misleading or untrue, and will be given a common-sense sanity check before going live; encourage underage consumption of cannabis or otherwise be aimed, directly or indirectly, to those under the legal age of consumption — for example, by using characters that kids would relate to or by using children as models; encourage unsafe or irresponsible use of cannabis (for example, by featuring a someone driving under the influence, which would be totally messed up); imply that consuming cannabis has therapeutic or health benefits; positively associate use of cannabis with dangerous or irresponsible activities or those that require care or skill the influence of cannabis would impair (like driving a car); show people consuming, smoking, or under the influence of cannabis; give the impression that the product is an alcoholic beverage; and/or encourage the use of cannabis for its intoxicating effects. Prohibited: giveaways, for example, as prizes, of cannabis or cannabis accessories; and/or ads for or relating to products containing Delta-8 or Delta-10.  **Hemp-derived CBD**:  Hemp-derived CBD products are not subject to the above cannabis advertising requirements. Instead, the following items apply to campaigns for hemp-derived CBD products: ads cannot make medical, health, or therapeutic claims or show people consuming or smoking CBD products; the product being advertised must be non-ingestible, and from legally-derived CBD; ads must be targeted only to states where CBD products are legal; and/or landing pages cannot sell or advertise other products or services prohibited by other Tumblr policies.  **Pharmaceuticals:** In general, ads for pharmaceutical and other health-related products and services should be scrupulously honest and direct, not make inaccurate or exaggerated claims, and not promote the product or service for a different use than it is intended or approved for by appropriate regulatory bodies. Applies to: prescription and non-prescription drugs, pharmaceutical companies, medical devices, and clinical trials; health and dietary supplements, and nutrition products; and/or health claims, particularly those about the treatment, prevention, or diagnosis of diseases and conditions.  URL: <https://www.tumblr.com/policy/en/global-advertising> | Any advertising related to the sale of cannabis or general promotion of cannabis brands must be (i) approved in advance by Tumblr, (ii) sold through our direct-sales team (i.e. not programmatic), and (iii) geo-targeted exclusively to people of legal consumption age in the above states (that’s 21+ in each case). Regardless of age-targeting, cannabis ads must not be designed, or appear to be designed, to appeal to under-age purchasers.  URL: <https://www.tumblr.com/policy/en/global-advertising> |
| **Twitch** | Prohibits content to buy or sell illegal drugs, firearms, or counterfeit goods. Prohibits activity that may endanger your life, lead to your physical harm, or encourage others to engage in physically harmful behavior is prohibited. What is not allowed is discussing or broadcasting any of these topics in a way that glorifies, promotes, or encourages these potentially dangerous behaviors. Examples: use of hard drugs; misuse of legal substances and substances not fit for human consumption (e.g., prescription drugs, whippets, tide pods); dangerous consumption of alcohol or other substances that lead to being incapacitated; and/or dangerous or distracted driving, including using a phone while driving and drunk driving. Usernames and display names created on Twitch may not include references to recreational drugs, hard drugs, and drug abuse, with exceptions for alcohol, tobacco, and marijuana.  URL: <https://safety.twitch.tv/s/article/Community-Guidelines?language=en_US> | **General guidelines**: Prohibited ad content includes: cannabis-related products, including vaping, delivery, and CBD; habit-forming drugs or drug-related paraphernalia, including tobacco products, e-cigarettes or vaping, nicotine, and similar products; herbal remedies or “miracle cure” products, or other products or services with questionable health claims; products, services, technology, or website content that violates applicable laws, or any content that features, encourages, offers, or solicits illegal activity; alcohol ads or ads from alcoholic beverage companies including “alcohol-free” and “non-alcoholic” beverage brands (US only); and/or products that are marketed to children through messaging, imagery, or targeting.  URL: <https://advertising.amazon.com/resources/ad-policy/twitch#prohibitedcontent> | Prohibited ad content includes products that are marketed to children through messaging, imagery, or targeting.  URL: <https://advertising.amazon.com/resources/ad-policy/twitch#prohibitedcontent> |
| **Twitter** | Prohibits use of Twitter for any unlawful purpose or in furtherance of illegal activities, including selling, buying, or facilitating transactions in illegal goods or services, as well as certain types of regulated goods or services (e.g., drugs and controlled substances).  URL: <https://help.twitter.com/en/rules-and-policies/regulated-goods-services> | **Drugs**: Prohibits the promotion of drugs and drug paraphernalia, including: illegal drugs, recreational and herbal drugs, accessories associated with drug use, drug dispensaries, and/or depictions of hard drug use.  **CBD**: Permits approved CBD topical advertisers to target the US. Advertisers: must be licensed by the appropriate authorities and pre-authorized by Twitter; may only promote non-ingestible, legally derived CBD topical products; may only target jurisdictions in which they are licensed to promote these products or services online; may not target Georgia, Idaho, Iowa, Mississippi, Missouri, Nebraska, Oklahoma, South Dakota, and Virginia; are responsible for complying with all laws and regulations; and may not target customers under age 21.  **Pharmaceuticals**: Permits ads for brick and mortar pharmacies, provided that they do not promote the online sale or refill of prescription drugs. These advertisers may only target the US, and are only allowed with prior authorization from Twitter. Ads from pharmaceutical manufacturers are permitted with restrictions.  URL: <https://business.twitter.com/en/help/ads-policies/ads-content-policies/drugs-and-drug-paraphernalia.html>  URL: <https://business.twitter.com/en/help/ads-policies/ads-content-policies/healthcare.html> | Prohibits knowingly marketing or advertising the following products and services to minors (<18): any controlled substance or paraphernalia; and/or drug paraphernalia.  URL: <https://business.twitter.com/en/help/ads-policies/ads-content-policies/prohibited-content-for-minors.html> |
| **YouTube** | Prohibits posts: aiming to directly sell, link to, or facilitate access to controlled narcotics and other drugs; making the sale of these items or facilitating the use of these services possible by posting links, email, phone number or other means to contact a seller directly; **displaying hard drug uses (n**on-educational content that shows the injection of intravenous drugs like heroin or huffing/sniffing glue); **making hard drugs (n**on-educational content that explains how to make drugs); depicting **minors using alcohol or drugs (s**howing minors drinking alcohol, using vaporizers, e-cigarettes, tobacco or marijuana, or misusing fireworks); or featuring drugs with the goal of selling them – if you're using links in your description to sell hard drugs, your channel will be terminated.  URL: <https://support.google.com/youtube/answer/9229611>  URL: [https://support.google.com/youtube/answer/2801964?hl=en#](https://support.google.com/youtube/answer/2801964?hl=en) | **Recreational drugs and drug-related content**: Content that promotes or features the sale, use, or abuse of illegal drugs, regulated legal drugs or substances, or other dangerous products is not suitable for advertising. Content promoting or glorifying drug usage, such as providing instructions on buying, making, selling, or finding illegal drugs or drug paraphernalia in order to encourage recreational usage should not appear. Some examples of content that fall into this category: sharing drug reviews and drug insights; tips or recommendations on recreational drug usage or creation, such as cannabis farming; reviews of cannabis coffee shops, head shops, dealers, dispensary tours, etc.; selling or buying drugs online or offline; and sharing links to drug purchasing sites or the physical addresses of drug purchasing locations.  This content can earn ad revenue: educational, humorous, or music-related references about recreational drugs or drug paraphernalia, where the intent is not to promote or glorify illegal drug usage; drug deals shown in gaming content; and documentary or journalistic reports portraying dramatized content using drugs – some examples of content that fall into this category: educational content about drugs or drug paraphernalia, such as the scientific effects of drug use or the history of drug trafficking; personal accounts of drug addiction recovery; music videos with fleeting depiction of drugs; gaming content displaying drug dealings; documentary or journalistic reports on the purchase, fabrication, usage, or distribution of drugs, such a story about a drug bust; and dramatized, documentary, or journalistic report including gaming scenes with consumption or usage (such as injection) of drugs. This content may earn limited or no ad revenue: non-educational and non-informational content focusing on illegal drug consumption (including injection) or creation, where the intent is not to promote or glorify illegal drug usage – some examples of content that fall into this category: dramatized content, including music and video games, showing recreational drug usage; and scenes of injecting drugs to get high in a scripted content. This content will earn no ad revenue: content promoting or glorifying drug usage, such as providing instructions on buying, making, selling, or finding illegal drugs or drug paraphernalia in order to encourage recreational usage – some examples of content that fall into this category: sharing drug reviews and drug insights; tips or recommendations on recreational drug usage or creation, such as cannabis farming; reviews of cannabis coffee shops, head shops, dealers, dispensary tours, etc.; and selling or buying drugs online or offline (sharing links to drug purchasing sites or the physical addresses of drug purchasing locations).  URL: <https://support.google.com/youtube/answer/6162278#Recreational_drugs>  URL: <https://support.google.com/youtube/answer/6162278?hl=en> | Prohibits content that endangers the emotional and physical well-being of minors (<18 in most countries/regions). May consider the following factors when deciding whether to age-restrict or remove content, depending on whether: the content promotes a product that contains drugs, nicotine, or a controlled substance; the upload is educational, documentary, scientific or artistic in nature; and there's any commentary discouraging the act (among others).  URL: <https://support.google.com/youtube/answer/2801964?hl=en&ref_topic=9282436>  URL: <https://support.google.com/youtube/answer/2801999?hl=en>  URL: [https://support.google.com/youtube/answer/2801964#zippy=%2Cage-restricted-content](%20https://support.google.com/youtube/answer/2801964#zippy=%2Cage-restricted-content   ) |
| *Notes*: * Assuming prescription drugs/pharmaceuticals may include medical marijuana. ** The Children's Online Privacy Protection Act dictates that the minimum age for social media use is 13 years old. Advertising to children under age 13 highly restricted, per the Guidelines for Responsible Advertising to Children issued by the Children's Advertising Review Unit. | | | |
